# Construction of knowledge constraints: a case study of 3D structural modeling

**DOI:** 10.1038/s41598-024-55115-4

**Published:** 2024-02-27

**Authors:** Cai Lu, Xinran Xu, Bingbin Zhang

**Affiliations:** 1 School of Information and Communication Engineering, University of Electronic and Science Technology of China, Chengdu, China; 2 School of Resources and Environment, University of Electronic and Science Technology of China, Chengdu, China

**Keywords:** 3D model, Knowledge reasoning, Interpretation, Complex geological structures, Geology, Tectonics

## Abstract

The uncertainty of structural interpretation complicates the practical production and application of data-driven complex geological structure modeling technology. Intelligent structural modeling excavates and extracts structural knowledge from structural interpretation through human–machine collaboration and combines structural interpretation to form a new model of complex structural modeling guided by knowledge. Specifically, we focus on utilizing knowledge rule reasoning technology to extract topological semantic knowledge from interpretive data and employ knowledge inference to derive structural constraint information from complex geological structure models, thus effectively constraining the 3D geological structure modeling process. To achieve this, we develop a rule-based knowledge inference system that derives theoretical models consistent with expert cognition from interpretive data and prior knowledge. Additionally, we represent the extracted knowledge as a topological semantic knowledge graph, which facilitates computer recognition and allows estimation of intersection lines during 3D geological modeling, resulting in the creation of accurate models. The applicability of our proposed method to various complex geological structures is validated through application tests using real-world data. Furthermore, our method effectively supports the realization of intelligent structure modeling in real working area.

## Introduction

Structural modeling is usually not the ultimate goal but supports the numerical and physical simulation of complex phenomena (such as seismic propagation and fluid migration), depth domain imaging, lithology interpretation, and reservoir modeling. The three-dimensional model of the underground structure visually shows the geometric shape and spatial relationship between underground geological interfaces and geological bodies, such as strata and faults^1–3^, Based on structural modeling, sequence and attribute modeling can be performed to directly support reserve calculations, well location deployment, and oil and gas development plan formulation. This is one of the most important tasks in underground resource exploration and development^4,5^. In these areas, the quality of seismic data is often poor, and the relationship between key stratum reflection and stratum contact in the seismic profiles is unclear^6^. The transformation of the multistage tectonic movement led to strong deformation of the rock mass, forming a very complex underground structure. Such cases often complicate the acquisition of high-quality structural interpretations, causing considerable uncertainty in the traditional data-driven structural modeling methods^7–9^. The presence of uncertainties can make it challenging to establish a direct link between the geometry of a 3D structural model and the corresponding geological interface^10,11^. Because of the high cost of obtaining interpretation data in actual structural modeling, only limited data can be obtained in a certain research area, which requires more expert experience and interpretation to construct a relatively accurate stratigraphic model^1,12,13^.

**Figure 1:**
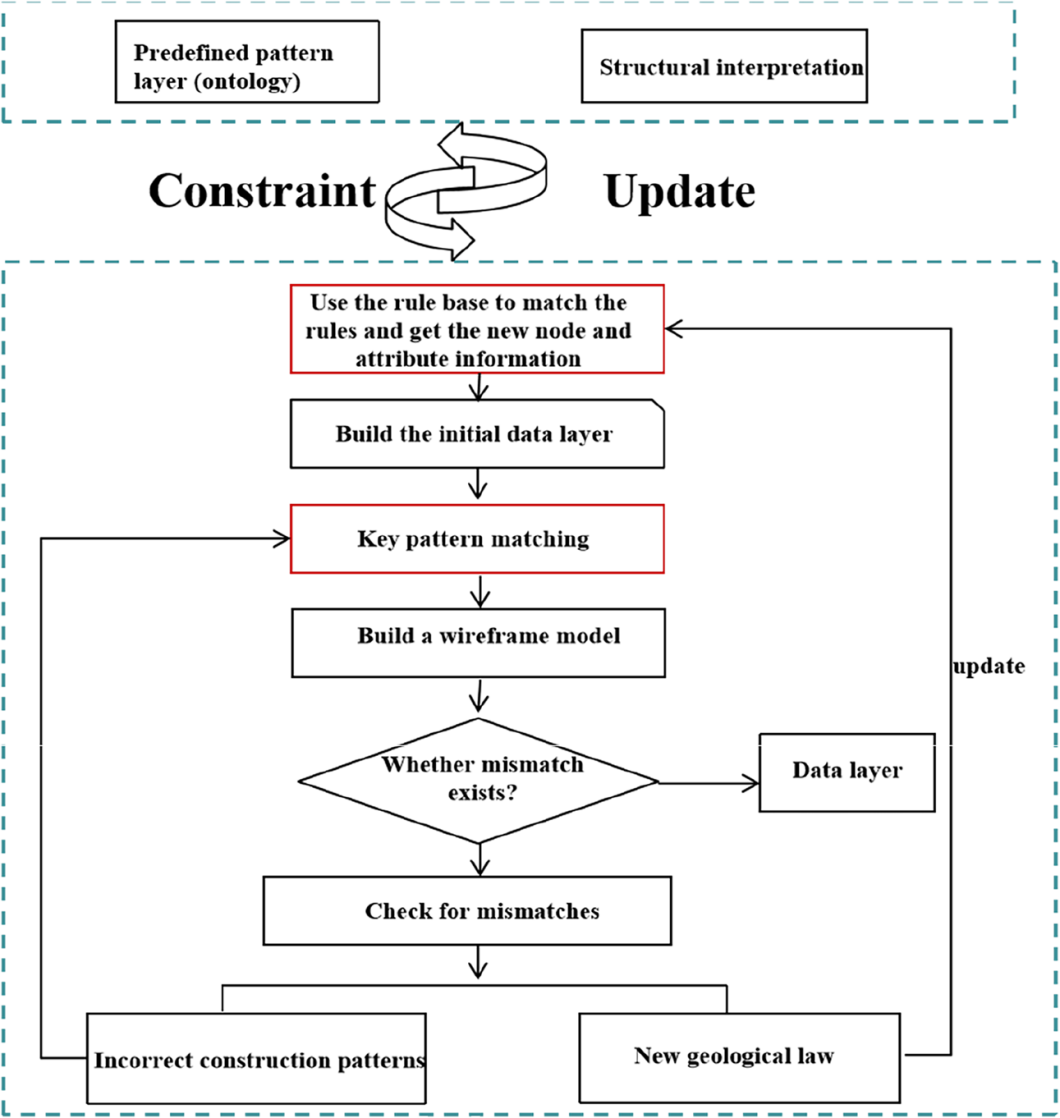
Knowledge constraint construction process.

Based on the above questions, Zhan et al. constructed the geometric constraints of a structural model through a knowledge graph, which they used to characterize the constraint relationship among knowledge. When the experts failed to comprehend the structural model, a quality assessment was conducted by modifying the knowledge graph to avoid repeated modeling operations^14^. From the graph perspective, knowledge graph is conceptual network and symbolic expression of the physical world. Its nodes represent entities in the real world, and the edges connected by entities represent the semantic links between entities^15,16^. Knowledge reasoning often involves overcoming two challenges: the difficulty of obtaining data, which results in sparse and uneven distribution of data samples, and the complexity of spatial relationships between structural elements^17^. These challenges complicate the accurate expression of geological structures using only text data, and the problem of missing structures is sometimes encountered. To address the above issues, this study proposes a process of constructing constraint information of complex geological structure modeling based on knowledge reasoning. The study aims to establish a large-scale structural modeling knowledge base, which is used to fuse the topological position relationship of geological surface space elements and provide technical support for 3D geological structure modeling technology, as shown in Fig. 1. The construction of a knowledge graph is divided into three stages: (1) conversion of geological data into constraints of a topology knowledge graph, (2) mining of entity and relationship information in geological data through knowledge reasoning, and (3) expert determination of the reliability of the knowledge graph using the wire-frame model.

This study makes the following contributions:

1. We propose a framework and process to obtain modeling constraint information by knowledge inference of complex geological structure models (Fig. 1), transform the knowledge of geological experts into knowledge graph data structures that can be recognized by computers, and represent them into wire frame models that can be recognized by experts.
2. We construct a common knowledge reasoning rule base in the field of structural modeling and introduce the semantic information of the geological structure into the topological network of the structural model.
3. We have demonstrated that our approach can effectively deal with real world work and avoid modifying the original data, but change the knowledge in the knowledge base, and improve the accuracy and robustness of the modeling by introducing expert knowledge.

The remainder of this article follows the following organizational structure. “Materials and methods” section briefly describes the relevant research methods of this study. In “Complex geological structure knowledge reasoning” section, the research methods and processes of constructing constraint knowledge of topological geological modeling through knowledge inference are introduced in detail. “Result” section shows the application of the proposed framework to a field case. “Discussion” section discusses the feasibility of the proposed framework in comparison with existing approaches. The last section will summarize the work of this study.

## Materials and methods

As far as we know, only Zhan et al.^14^ have introduced knowledge into the study of structural modeling at present, and there is no relevant study on the constraint information of knowledge inference to construct modeling. In this section we will discuss the following two types of research work that are relevant to this paper: knowledge reasoning and explicit modeling^18^.

### Explicit structural modeling

**Figure 2:**
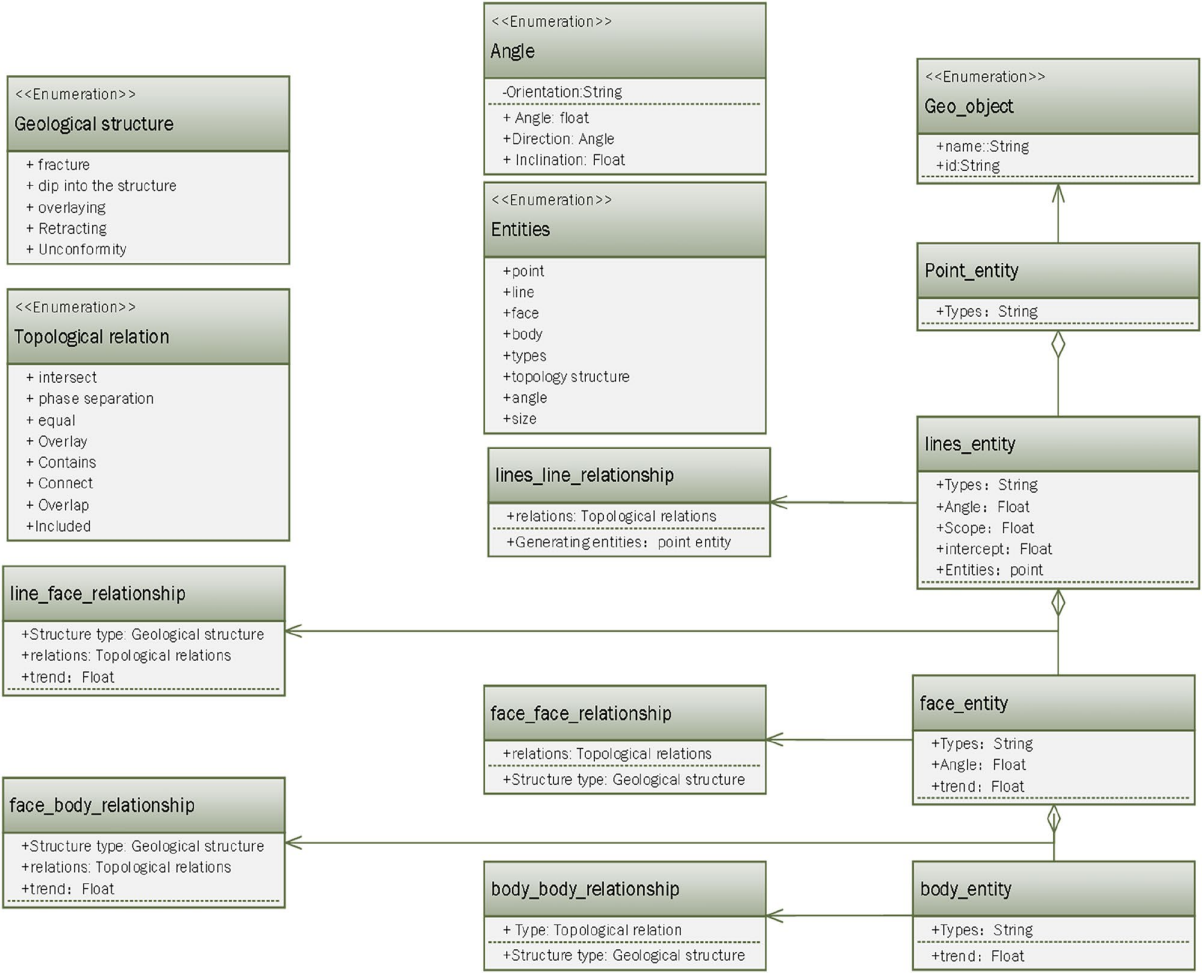
Knowledge constraint construction process.

Explicit interactive modeling is a classical 3D modeling method to reconstruct 3D geological structures using sparse data. When modeling complex regions containing various types of geological structures, the limitations of using a single method show up. In display modeling, people introduce the workflow of building closed geological models by introducing multi-source data information to constrain 3D geological models. First, all geological surfaces are reconstructed, and then the intersections between them are found by cutting each other under constraints. Most of the subsequent explicit modeling approaches use this workflow. Display modeling allows a large number of interactive modifications, adding appropriate underground control constraints according to the experience of the modeler, but the amount of interaction is very large and prone to topological contradictions^19^. The square deterministic modeling of structural modeling mainly includes the following methods, namely reservoir seismology methods^20^, reservoir sedimentological methods^21^ and kriging interpolation methods^22^. It includes some discrete techniques commonly used in software^23–26^. Yan et al. shown that spatial explicit models produce better results than non-spatial models, thus showing that space is indeed special in terms of summary^27^. In order to build a more reasonable 3D geological structure model, but now lack of understanding of the specific structure. In display modeling, where it is difficult to choose a way to integrate all types of information, geological histories are used to combine multiple scalar fields, merely showing the geological interfaces cutting each other. We focus on introducing knowledge reasoning into structural modeling to construct structural models, and introducing earth scientists' cognition into display modeling to provide more comprehensive and intuitive structural models. We provide a workflow to construct modeling constraints by knowledge reasoning, and improve the stability and efficiency of display modeling by introducing knowledge reasoning. Figure 2 provides constraint information for the pattern layer.

### Knowledge reasoning of model constraints

The model constraint can be represented by knowledge graph in data form the construction of the knowledge constraints of the structural model starts from the application field and determines the scope of knowledge constraints^28^. The key to constructing the data layer of the knowledge graph is knowledge reasoning^29^. New insight is obtained through knowledge reasoning, and the given knowledge graph is inferred based on expert knowledge to determine whether it conforms to cognition for updating the rule base of the pattern layer of the knowledge graph.

The target world can be described based on the relationships between entities. Based on this information, the data are not sufficiently meaningful. Relevant data were combined to form the information. Semantics comprise two components: data and relevance. When describing a structural model, the semantic description encompasses both low-level features, such as geometric properties, and high-level features, such as logical relationships between structural elements. By utilizing basic semantic entities, the structural geological model is divided into geometric units, and their spatial topological relationships are described through the relationships between geometric elements. The semantic reasoning between spatial topological geometries yields the construction process of a knowledge graph^30^.

The semantic entities in the modeling knowledge graph refer to geometric objects. Their basic semantic entities can be divided into four types: point (0-cell), line (1-cell), surface (2-cell), and body (3-cell). Relation refers to the topological geometric, positional, and compositional relationships between two entities (including the relationships between target entities). An attribute refers to the position and closure of geometric objects in structural modeling^14^.

#### Definition 1

(*Topological semantic knowledge graph*) The creation and update of topological semantic knowledge graphs can be recorded using meta-knowledge, thereby enabling evolution analysis and traceability of complex geological structural knowledge.

1 $$ GeohfKG = < E,V,\{ [P_{T} ,Ti]|Ti_{O} ,[P_{L} ,L]|L_{O} \} |R_{Ti,L} > \leftrightarrow GeoMetaK, $$

where $E,V$ represent the basic elements of the topological semantic knowledge graph. Usually, they are expressed in the form of “head entity, relationship between entities, tail entity”.$GeoMetaK$ represents the meta-knowledge of the topological semantic knowledge graph., It is usually used to indicate the updating of knowledge, $P_{T}$ and $P_{L}$ are used to represent the temporal and spatial relationships of topological semantic knowledge graphs, such as the deposition time order of the interpretation data and the topological spatial location association.

Introducing a knowledge-reasoning algorithm into a knowledge graph to constrain its construction and obtain accurate data samples is necessary. In constructing a topological structure knowledge graph, it is very important to determine the relationship between structural elements. Burns et al. introduced a technique for representing the geological topological relations using a network diagram, in which node entities denote spatial elements and edges indicate the topological connections between them^31^. Based on the above ideas, we used the hierarchical network to obtain the computer representation of the topological structure knowledge graph. The topological structure information fully represented the geometric and structural features of the structural geological model.

## Complex geological structure knowledge reasoning

### Knowledge reasoning rule base construction

The pattern layer of a knowledge graph consists of defining entities, relationships, and attributes, as well as building the knowledge graph’s rule base. Structural geology has a long history. Numerous rules and a lot of information regarding the genesis and regularity of movement of geological entities have been formed. Some of these rules can be summarized to form knowledge.

**Figure 3:**
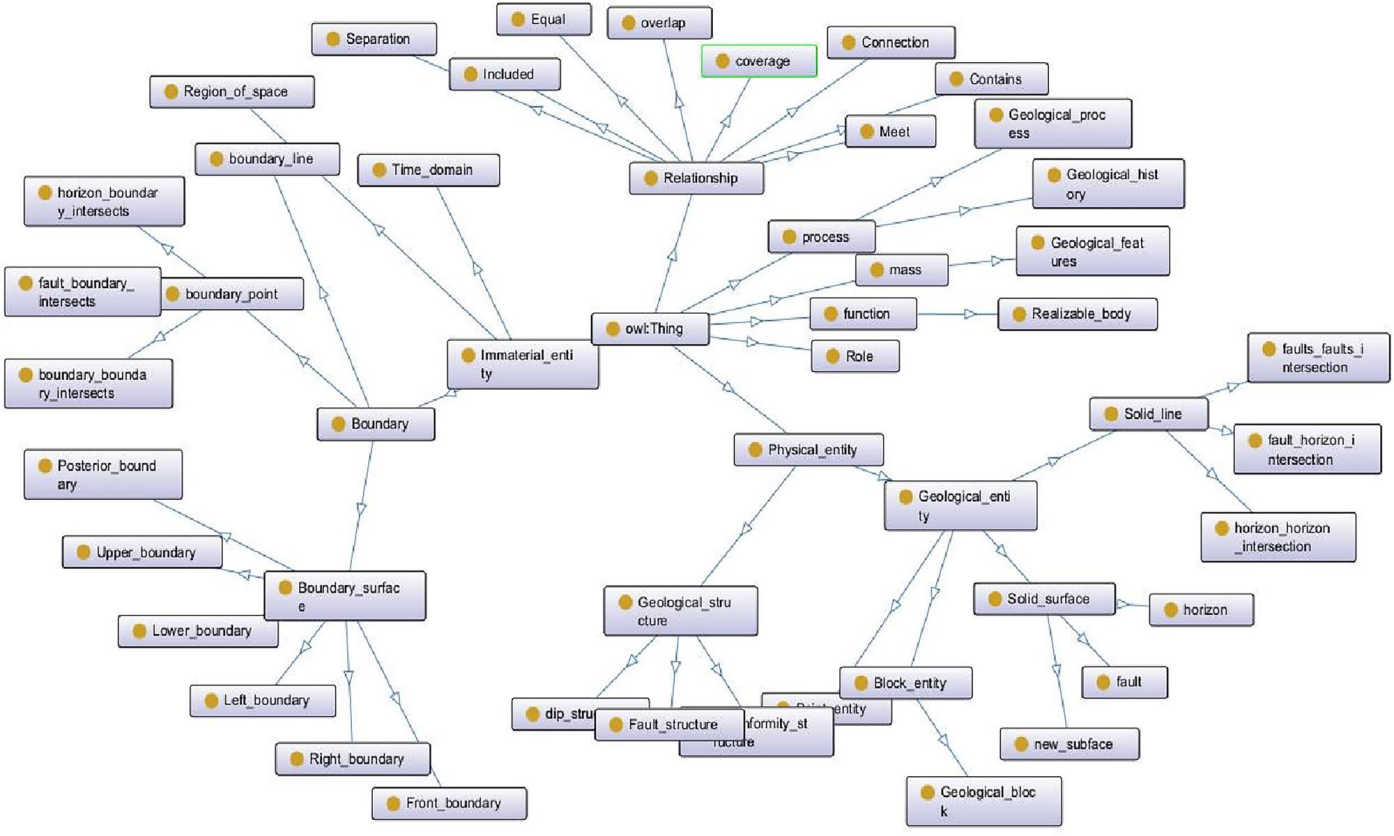
Structural geology knowledge ontology graph.

**Figure 4:**
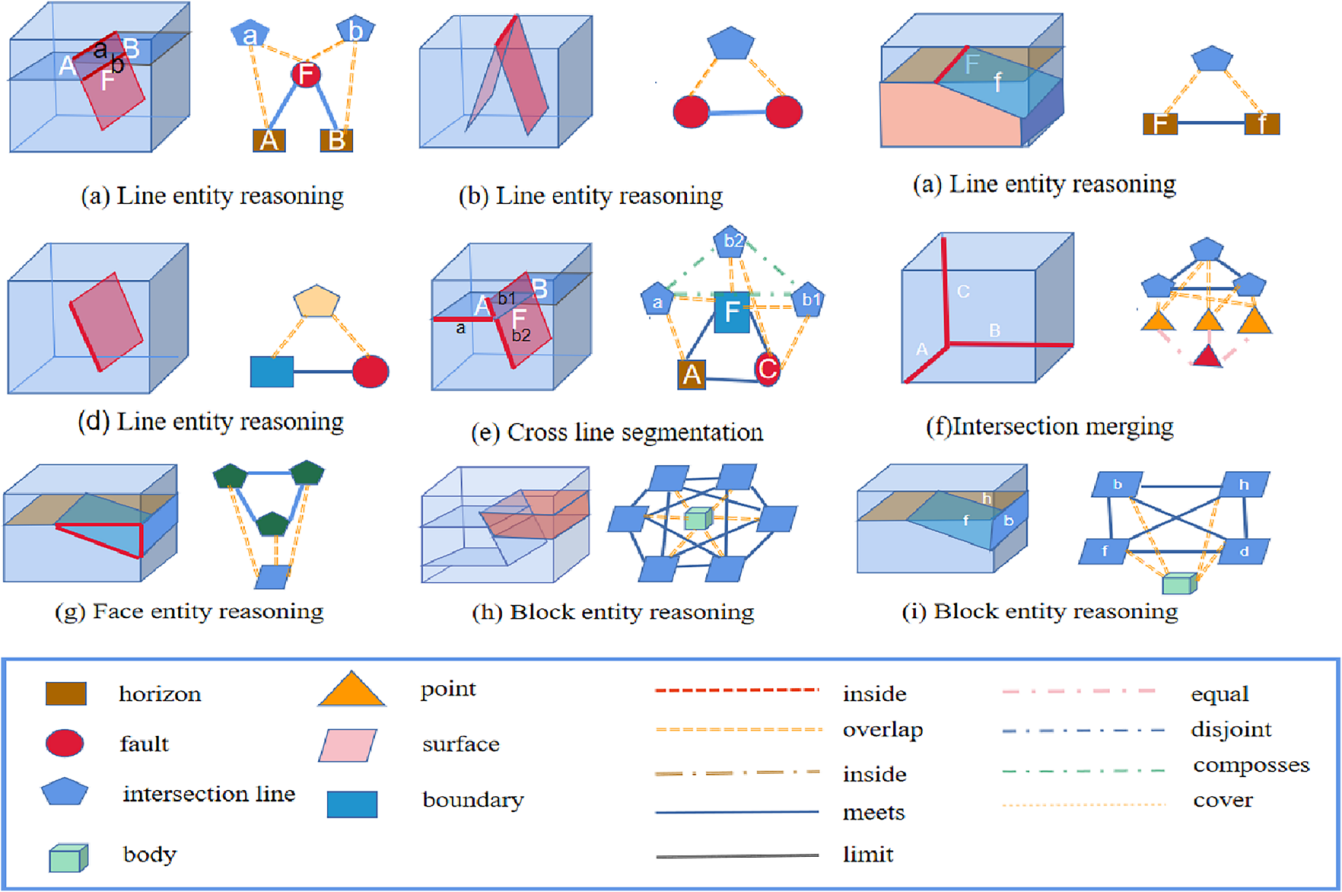
Topological rule base of knowledge reasoning, initial predefined geological structure rule base (geological structure diagram), including six common reasoning modes and subgraph reasoning query diagram; the geometric topology rule base (geometric structure diagram) contains common geometric reasoning patterns.

Therefore, according to the definition of a knowledge graph in the construction of a complex-structure knowledge graph, and the schema layer and rule base summary of knowledge constraints are shown in Figs. 3 and 4.

The basic form of the production rule base in this article is

2 $$ R_{{\text{k}}} : = \mathop {OR}\limits_{j = 1}^{n} \left( {\mathop {AND}\limits_{j = 1}^{m} E_{ijk} } \right) \to C_{k} \quad m \ge 1,n \ge 1. $$

Among them, $R$ it represents the number of rules, k is the k rule, which represents the obtained rule conclusion, and the extraction of the knowledge graph data layer is realized through logical rules. Where $E$ and $C$ represent the conditions and conclusions of rule reasoning. The pattern layer of a knowledge graph consists of defining entities, relationships, and attributes, as well as building the knowledge graph’s rule base. Structural geology has a long history. Numerous rules and a lot of information regarding the genesis and regularity of movement of geological entities have been formed. Some of these rules can be summarized to form knowledge. Therefore, according to the definition of a knowledge graph in the construction of a complex-structure knowledge graph, the rule base of knowledge reasoning is summarized as shown in Fig. 4.

In the initial rule base, we first added nine separate geological structure rule patterns as the initial state and then used the geological constraint rule base (prior knowledge) to match the common structural patterns. For parts that match, we inferred the interpretation of the corresponding geological structure to be geologically reasonable. In the construction of model knowledge reasoning, the focus was on geometric topology knowledge graphs from the perspective of computer geometry and knowledge graph reasoning. In the new reasoning process, we focused on two possibilities: (1) different structural superposition and superposition methods since exhausting all geological structure rules is difficult, and (2) predefined rule errors (prior knowledge errors), as shown in Fig. 4. Experts must determine whether new rules need to be added or whether existing rules are suitable. In the process, the inference rule base is gradually improved and optimized.

The topological structure knowledge graph can be used to represent and describe various geological structural models, including fractures, intrusions, and unconformities. By representing these construction patterns as nodes and using edges to describe the relationships between them, a topology knowledge graph can be constructed. In the knowledge graph, the relationship between different nodes can be expressed as a topological relationship, which can more accurately represent the topological relationship in the geological structure and automatically perform topological inspection and constraints during the modeling process to ensure the correctness of the model.

**Figure 5:**
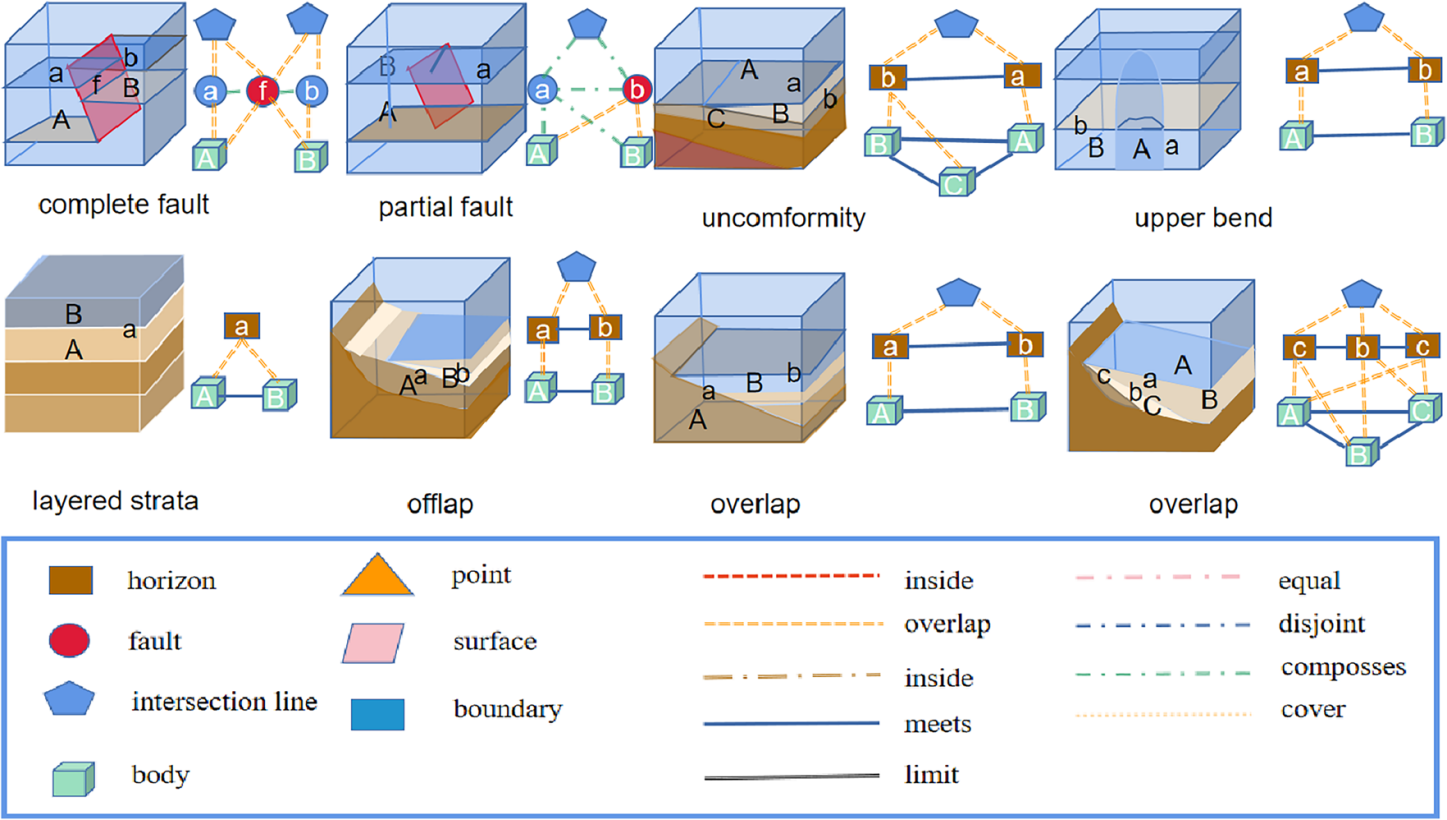
Lists common geological structure models, such as faults, intrusions, and unconformity structures. Most of the common geological structures exist in fault structures and unconformity structures. In the proposed rules, the above models can be expressed in the form of a knowledge graph.

The geological processes and structural types covered in the rule base are extensive, covering common types of geological structures such as faults, folds, and intrusions and geological structures of different scales, such as large-scale topography and small-scale rock structures. We described the universality of the rule base from three perspectives. (1) The first perspective involves the types of covering tectonic patterns; we listed the types of tectonic patterns contained in the rule base, such as faults, intrusions, and unconformity structures, as shown in Fig. 5. This rule base can cover the most common construction patterns in graphs. (2) The second perspective is the spatial distribution of cover tectonic models. In addition to the types of cover tectonic models, we considered the distribution of these tectonic models in geological space, which can describe various tectonic models in different geological periods and regions. (3) The third perspective includes the complexity of the cover structure model; the complexities of the geological structure models differ. Certain simple models may be relatively easy to describe and identify, whereas others may require more rules to describe. In the subsequent simulation, the given rules were used to achieve a more complex model (Fig. 5). Therefore, our rule base can cover and handle construction patterns with different complexities.

### Knowledge reasoning for constraints of geological structure modeling

Knowledge reasoning, which can accurately connect to structural modeling, is the main technology used to build a knowledge graph in the field of structural modeling. The aim of constructing a knowledge graph is to obtain expert knowledge from seismic interpretation data and structured and semi-structured data from seismic interpretation data, such as coordinate data of the horizon and fault plane, intersection information of the horizon and fault plane, and the extraction of geological entities, spatial relations, semantic relations, and sedimentary relations. Furthermore, we determine whether the number of rules is finite or countable. Finally, a complete knowledge graph of the structural model was obtained by combining the knowledge reasoning of the spatial relations. In structural modeling, knowledge reasoning can combine spatial relations to achieve a more accurate reasoning process. For example, the relationship between geological entities can be inferred based on their relative positions and intersections in space.

**Figure 6:**
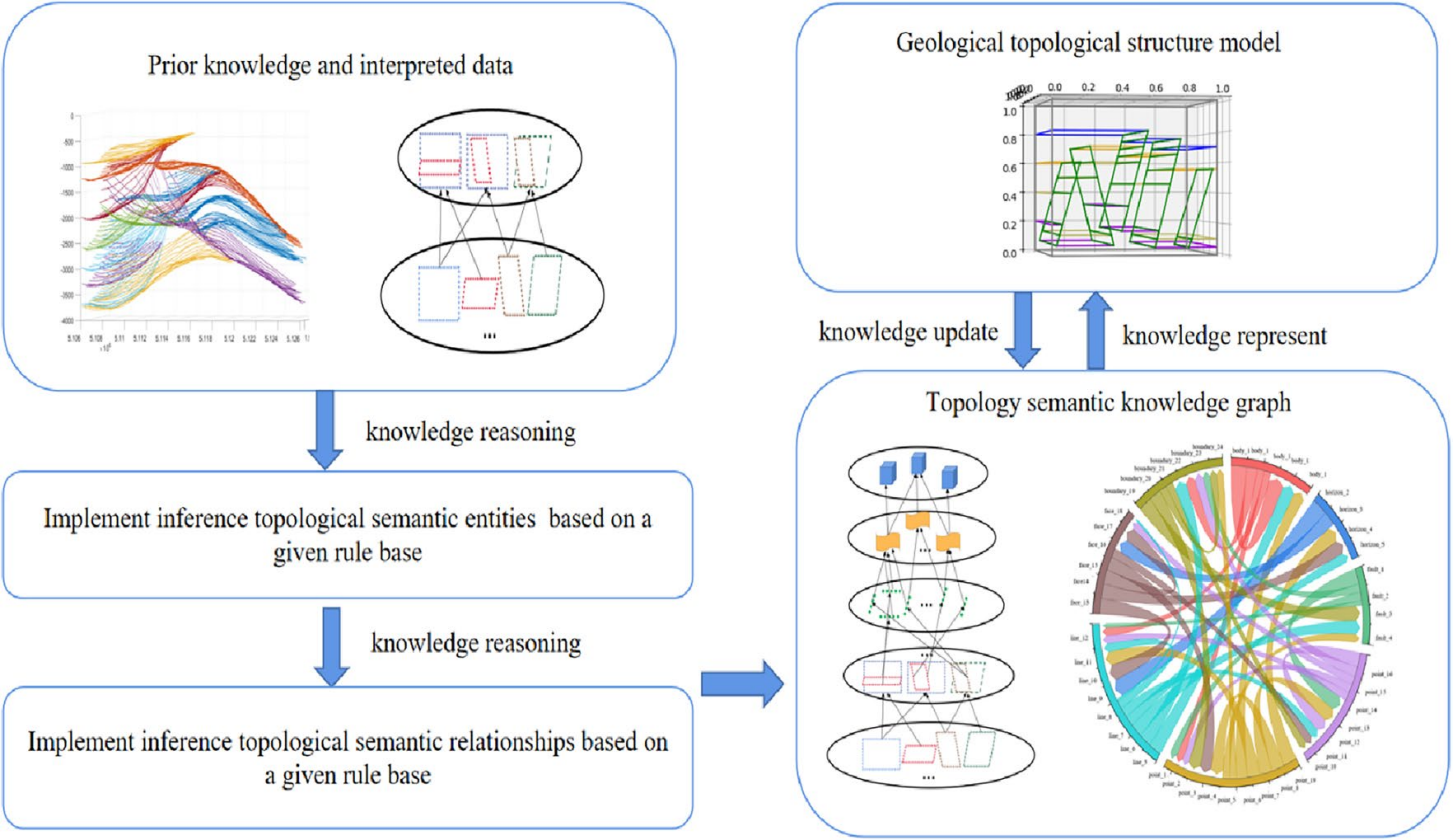
Complete process of knowledge reasoning can be divided into three parts: transforming geological data into geological constraints that can be read by computer; based on the constructed knowledge base, the knowledge graph is constructed via the knowledge reasoning algorithm. The knowledge graph is represented as a wireframe model to allow expert knowledge to participate in the knowledge graph update.

Reasoning constraints in the knowledge graph are realized through the rules of knowledge reasoning. The process of knowledge reasoning is as follows. The specific reasoning process is shown in Fig. 6. The aim of knowledge reasoning is to make human knowledge comprehensible to computers, construct a geometric topology knowledge graph, and guide 3D geological structure modeling. In our input, the effective horizon data is $H_{i} = \{ h_{{i_{1} }} ,h_{{i_{m} }} ,....,h_{ia} \}$, where $h_{{i_{m} }}$ represents the horizon interpretation data at the m point of the $i$ section, $M \le a$, $a$ is the number of horizon. And the effective fault data is $F_{j} = \{ f_{{j_{1} }} ,f_{{j_{n} }} ,...,f_{{j_{b} }} \}$ where the $f_{{j_{n} }}$ represents the fault interpretation data at the nth point of the j section, 1 $\le {\text{n}}\le {\text{b}}$, b is the number of fault. the quantity of a and b is determined according to the specific work area.

To facilitate processing, we modeled the input prior knowledge as an adjacency matrix. According to the prior knowledge of geological structure (the intersection information of plane and fault plane) obtained from the perspective of geological experts, the prior information is represented by $G_{1} = (E,V)$, where $E = \{ GeoMetaK,E_{e\_g} \}$,

3 $$ GeoMetaK = \left[ {\begin{array}{*{20}c} {e_{11} } & {e_{12} } & \cdots & {e_{1(m + n)} } \\ {e_{21} } & {e_{22} } & \cdots & {e_{2(m + n)} } \\ \vdots & \vdots & \ddots & \vdots \\ {e_{(m + n)1} } & {e_{(m + n)2} } & \cdots & {e_{(m + n)(m + n)} } \\ \end{array} } \right], $$

where $$e_{ij} = 1$$ indicates that the $$i{\text{-}}th$$ face and the *j*th face have an intersection relationship, $$GeoMetaK = \{ E_{F} ,E{}_{H},V\}$$$$E_{F}$$ and $$E_{H}$$ represent the fault plane and horizon plane entity respectively.

And $(m + n)(m + n)$ represents the number of intersection relations between horizon planes and fault planes calculated by the bounding box method as our meta-knowledge. $e_{ij} = 0$ indicates that there is no intersection relationship. And the $E_{e\_g}$ represents the point, line, surface, block semantic entity that interprets the data generated, and its specific form is $E_{e\_g} = \{ e_{{p_{1} }} ,e_{{p_{2} }} ,...,e_{{p_{n\_p} }} ,e_{{l_{1} }} ,e_{{l_{2} }} ,...,e_{{l_{n\_l} }} ,e_{{f_{1} }} ,e_{{f_{2} }} ,...,e_{{f_{n\_f} }} ,e_{{l_{1} }} ,e_{{b_{2} }} ,...,e_{{b_{n\_b} }} \}$ where $n\_p$, $n\_l$, $n\_f$ and $n\_b$ represent the number of the topological semantic entities of the generated point, line, surface and block entities respectively. What’s more $V = \{ V_{meet} ,V_{overlap} ,V_{inside} ,V_{disjoin} ,V_{{{\text{cov}} er}} ,V_{compose} ,V_{equal} ,V_{\lim ot} \}$. The following tables of the above edges represent the topological position relationships between entities respectively, represents the intersection information of prior knowledge (1 represents intersection, and 0 represents non-intersection) to construct the adjacency matrix of prior knowledge. Through the inference of rule base, the transformation of geological structure information to the topological relationship is realized, and the language (i.e., the form of adjacency matrix) that can be read by a computer is c-ombined to create conditions for the subsequent sub-graph inference. The pseudo-code of knowledge reasoning related to algorithm 1 based on the rule base is as Table 1.

**Table 1:**
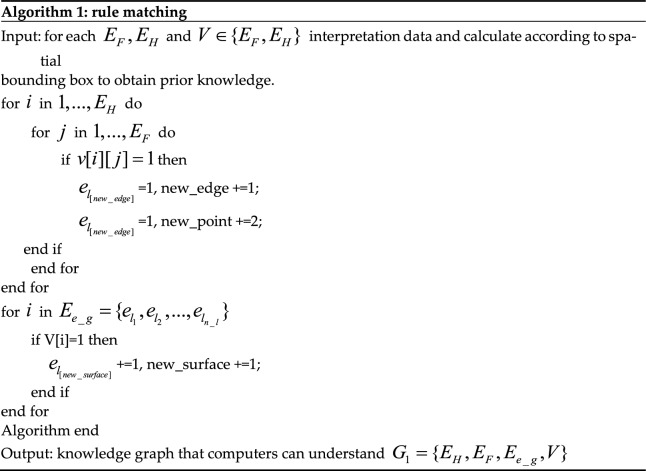
The algorithm of rule matching.

In the first stage of reasoning, prior human knowledge is combined with data that the computer can recognize, and computer cognition is realized by matching the corresponding rule base.

After obtaining the basic topological entities and relationship information of the knowledge graph, pattern matching in the knowledge base is carried out through sub-graph matching research. The planar entities and block entities existing in the knowledge graph are mined by graph isomorphism matching, and the entities and relationship information conforming to the geological structure are obtained by approximate subgraph matching. We studied the problem of sub-graph matching for knowledge graph. Specifically, given a query graph $G_{q} = \{ E_{query} ,V_{query} \}$ and data graph $G_{d} = \{ E_{data} ,V_{data} \}$ the sub-graph matching problem refers to obtaining all the data sub-graphs in $G_{2}$ that is isomorphic to $G_{3}$ to determine the new entity information in the knowledge graph. Through the study of each algorithm, we selected the VF2 algorithm with block efficiency and speed. The corresponding pseudo-code is as Table 2.

**Table 2:**
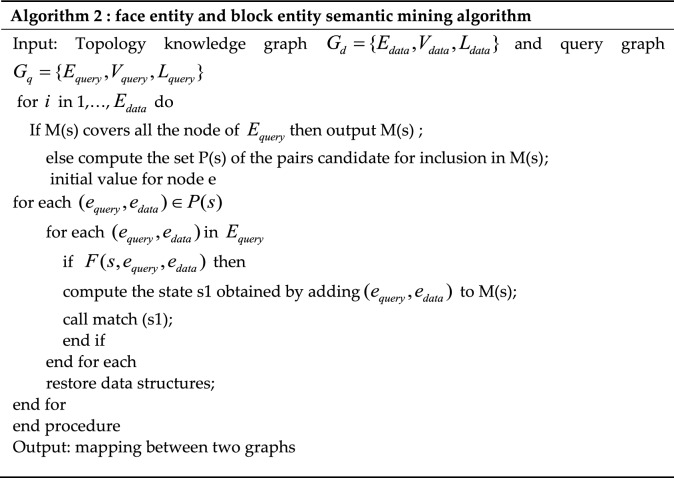
The algorithm of mining algorithm.

Generally, mapping M is expressed as the mapping of the node pair $(e_{query} ,e_{data} )$ ($e_{query} \in G_{q}$ and $e_{data} \in G_{d}$) each of which represents the mapping of node $e_{query}$ of $G_{q}$ and $e_{data}$ of $G_{d}$, $M \in G_{d} \times G_{q}$ the function $F(s,e_{query} ,e_{data} )$ is a feasibility function that simultaneously increases the comparison of node and edge labels.

The return value is a Boolean value used to prune the search tree. At the same time, it can elimiate the situation where the two graphs can be isomorphic but the final matching result cannot be obtained, which is used to reduce the number of state spaces. P (s) represents the set of all pairs of nodes to be matched. The VF2 algorithm is used to match the isomorphism graph, and a knowledge graph (face and block entities) that matches the given rule pattern is obtained. The VF2 algorithm cannot directly query the number of isomorphic sub-graphs; however, this function can be realized by modifying the VF2 algorithm. Specifically, based on the VF2 algorithm, each matched node pair can be marked, and unmatched node pairs can then be searched. Each time a new match is found, the marked node pairs are removed from the current search to query the number of isomorphic sub-graphs.

**Figure 7:**
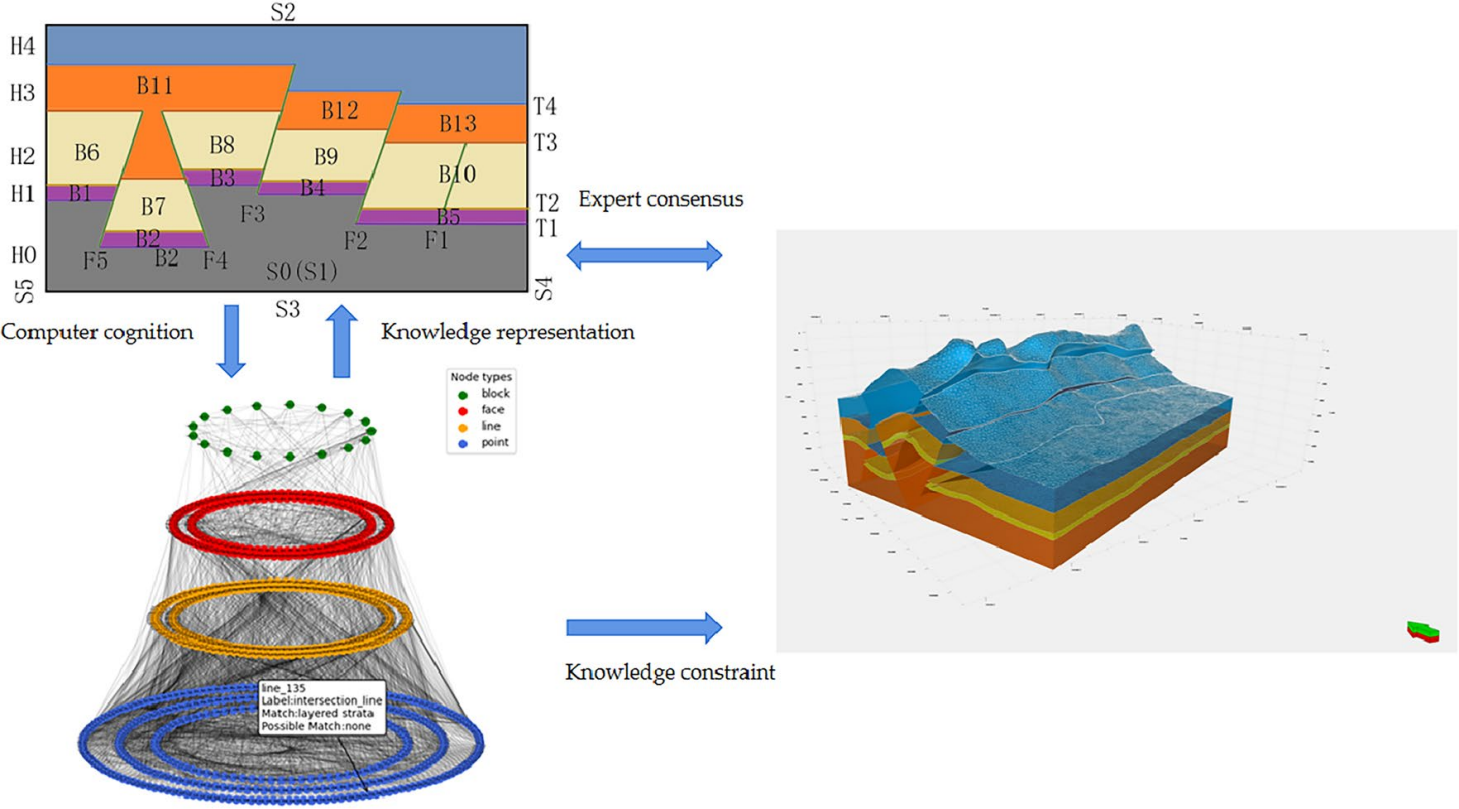
The whole knowledge reasoning ultimately participates in the workflow of the construction modeling.

The specific process uses Algorithm 1 to realize rule matching and converts prior human knowledge into structured data on the computer with the help of prior rules to guide subsequent subgraph isomorphism matching. Through the modified sub-graph matching model, all sub-graphs that are isomorphic to the graph are traversed through sub-graph isomorphism to infer all surface entities and block entity knowledge and construct a complete knowledge graph. The quality of a knowledge graph obtained through knowledge reasoning is typically not guaranteed; therefore, it is added to the knowledge base. Before the process of quality evaluation is required, the wire-frame model is used in construction modeling to evaluate the quality of the knowledge graph. As shown in Fig. 7, when the constructed knowledge graph does not conform to expert cognition, the error information in the knowledge graph is modified and queried using subgraph matching to identify incorrect subgraph information in the knowledge graph. Quality assessment is the process of measuring and evaluating the credibility of new knowledge before it is added to a knowledge base to eliminate errors or conflicts.

## Result

Figure 7 shows the workflow of knowledge reasoning construction modeling constraints participating in construction modeling. Firstly, the knowledge graph is represented as a wire-frame model to determine whether there are errors in the knowledge graph and realize the editing of the knowledge graph data. Secondly, the knowledge graph obtained by knowledge reasoning is a data format that can be recognized by the computer to constrain the boundary conditions of the construction model. Finally, the wire-frame model and the final construction model are consistent with expert knowledge.

**Figure 8:**
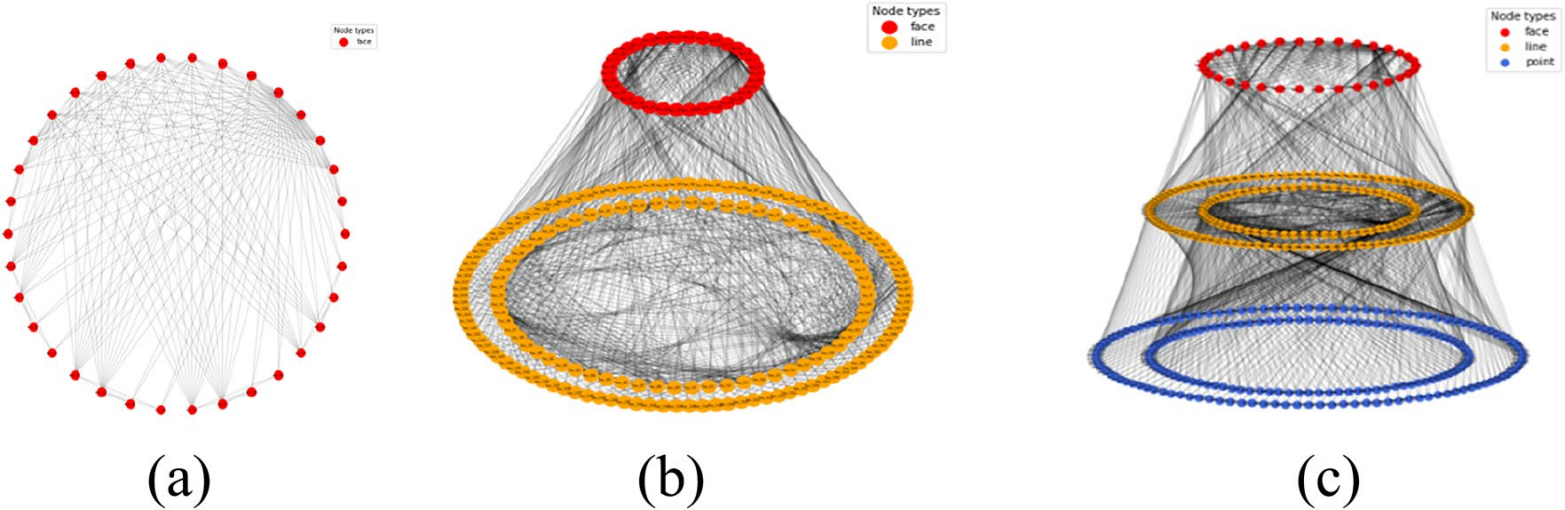
The process of point entity and line entity in the topological geometric knowledge graph obtained by rule base matching in the first stage of knowledge reasoning, which is divided into two steps. (**a**) Is prior knowledge, and geological experts input the intersection relationship between horizon surface and fault surface; (**b**) the topological position relationship between the line entity and the entity is obtained by matching the prior knowledge through the rule base; and (**c**) is the point entity obtained by the rule base matching for the intersection of line entities, and the entities with the same attributes are merged through knowledge fusion to complete the first stage of knowledge reasoning task.

**Figure 9:**
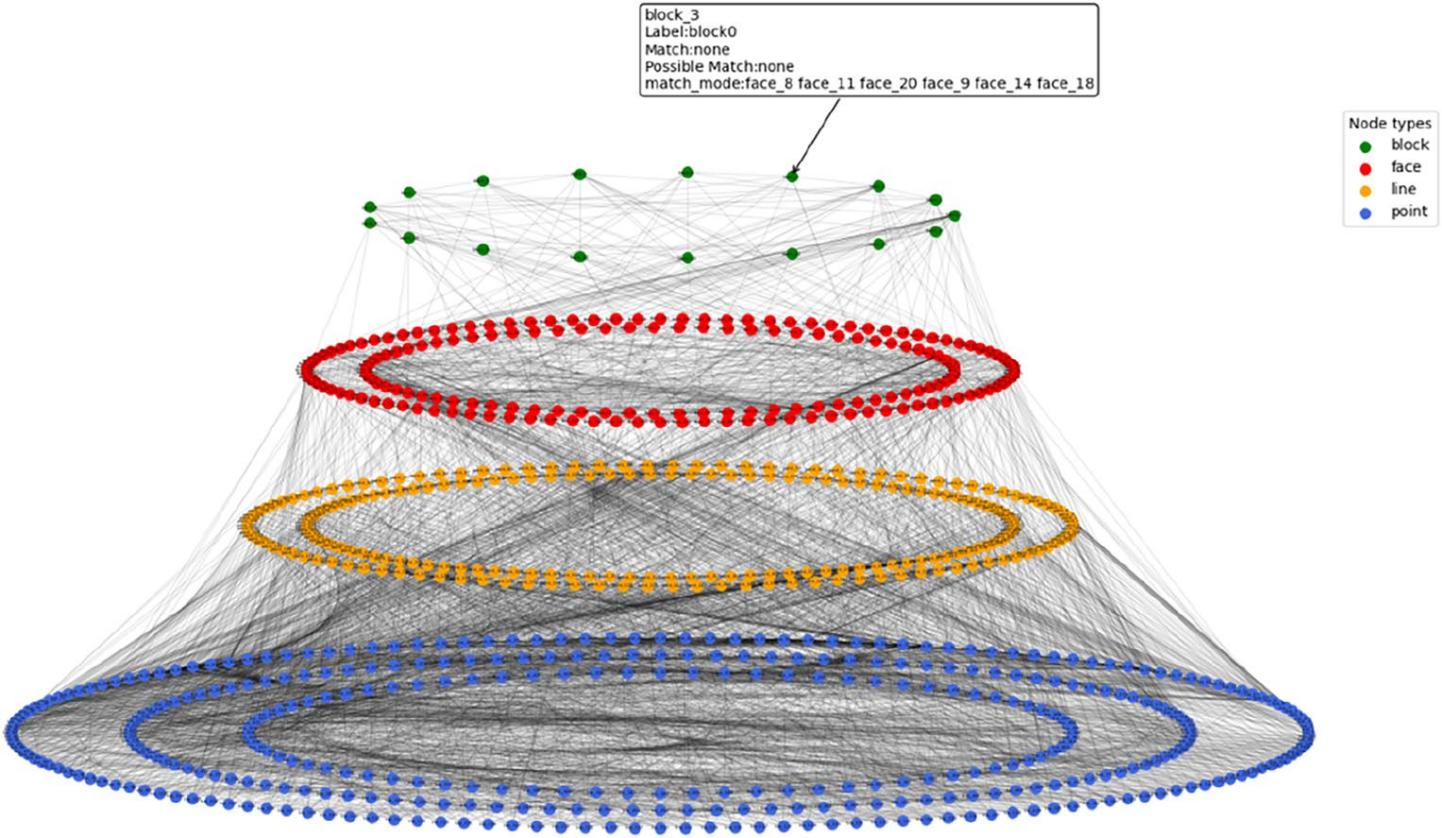
Second stage of knowledge reasoning, and the face entity and block entity obtained in the previous stage are used to obtain the subgraph isomorphic to the given graph model by subgraph isomorphism matching, and the matching entity and topological position relationship are recorded, to obtain the updated pure topological space knowledge graph.

**Figure 10:**
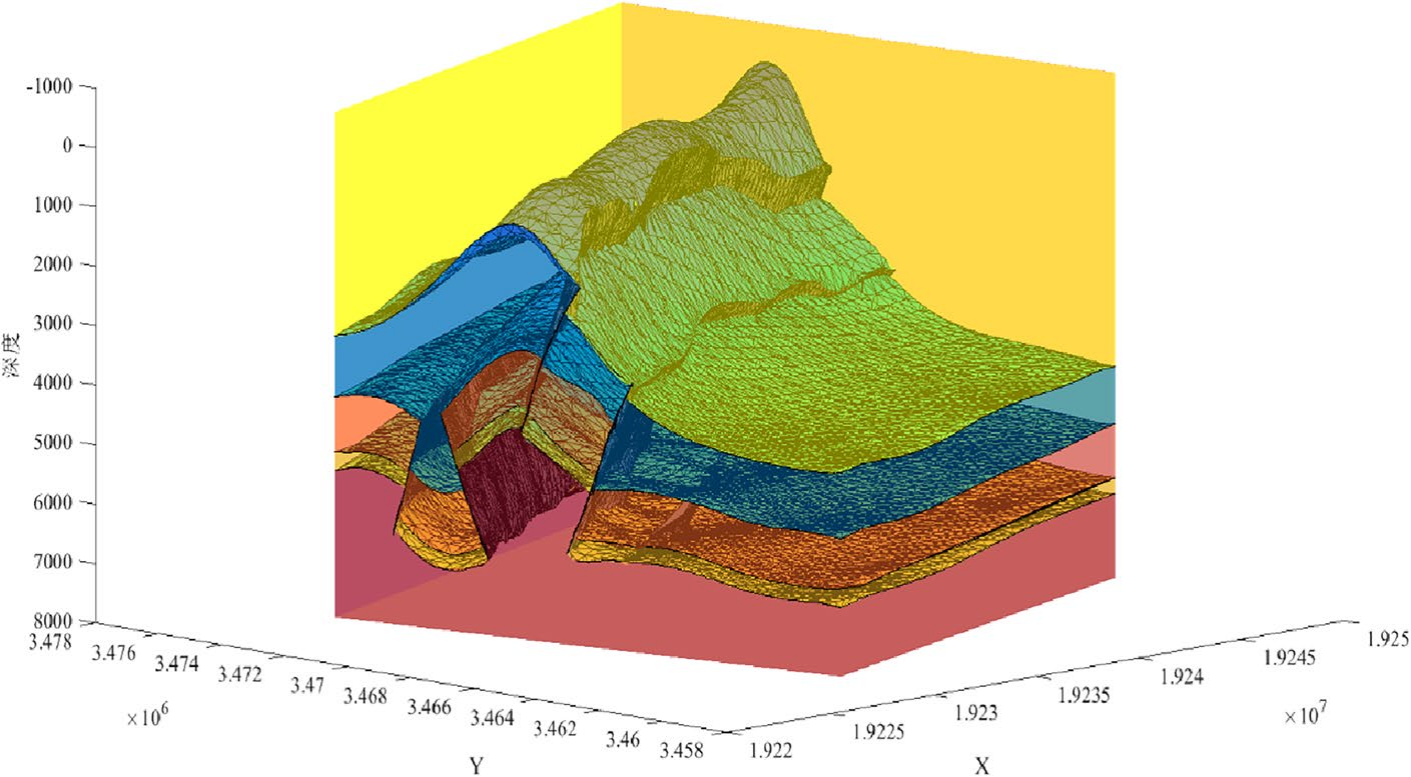
Closed geological body in the actual work area of three-dimensional geological structure under the constraint of knowledge graph.

In the construction process of knowledge reasoning in the knowledge graph, the entities and relationships in Fig. 8b,c are obtained through the first stage of knowledge reasoning using prior knowledge (Fig. 8a) (where prior knowledge is used to judge the intersection relationship between the horizon surface and fault surface by interpreting the data). Here, entities refer to the intersection line and intersection point entities obtained from prior knowledge, as well as the topological semantic relationships between entities. Rule matching in the library was used to realize the reasoning of geological knowledge data to topological structure data, and a preliminary knowledge graph was constructed. In the second stage of knowledge reasoning, the hidden entity and relationship information in the knowledge graph are continuously enriched, and all sub-graphs that are isomorphic to the query sub-graph are obtained through the sub-graph isomorphism query. As shown in Fig. 9, the hidden surface entity and geological block entity knowledge (third and fourth layers) were obtained by reasoning. The knowledge graph obtained in this study does not contain prior knowledge of the initial definition. It is a purely topological semantic knowledge graph. The intersection point, intersection line, closed surface composed of an intersection line, and closed block entity composed of a surface were mined from prior interpretation data. The knowledge graph obtained by knowledge inference is used as the modeling constraint to estimate the boundary conditions of the constructed model[5]and get the final structural modeling,as show in Fig. 10.

## Discussion

### Practical application of knowledge reasoning

**Figure 11:**
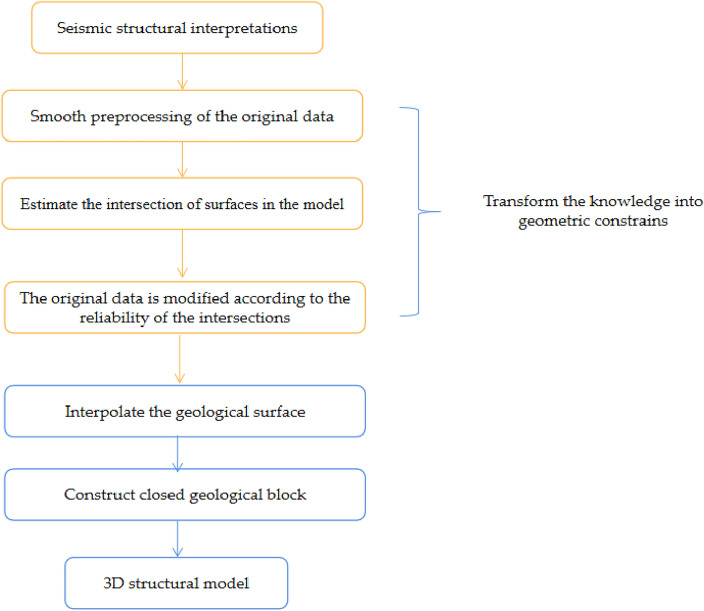
Complete process of knowledge reasoning constrained 3D construction modeling. It can be divided into two parts: knowledge reasoning participates in intersection estimation and three-dimensional geological body surface interpolation reconstruction closed three-dimensional geological body.

The constructed geometric constraint knowledge graph can be used to constrain 3D geological structure modeling^5^. The specific construction process is shown in Fig. 11. Knowledge graph technology is used to construct knowledge graphs in the field of geology so that geologists can query and analyze geological data and identify geological laws and evolution trends. According to the geological knowledge in the knowledge graph, the model was constrained and optimized. The constraint relationship in the knowledge graph is used to specify the topological relationship and geometric properties of the intersection line to improve the accuracy and controllability of the intersection line, provide the geometric constraints required for surface reconstruction, and ensure that the surface is as smooth as possible, while satisfying the geological structural characteristics. The optimized geological model was more consistent with the actual geological conditions.

In the subsequent modeling process, the topological structure knowledge graph is integrated into the boundary feature extraction, and then the boundary features are used as surface topology semantic constraints, and the morphological features are used as surface geometry semantic constraints, combined with multi-source information abstraction such as stratigraphic interpretation point clouds and well layered data. It is a multi-constraint surface regression model. By building a rectangular grid, based on the spatial autoregressive neural network, the fitting error and smoothing error are used as loss functions to solve the model, and then construct a model with high fitting degree, good smoothness and accurate morphological characteristics.

$$ \begin{aligned} & {\begin{aligned} \mathop {\arg \min }\limits_{{w_{i}^{{x_{{_{TL} }} }} ,w_{i}^{{z_{{_{TL} }} }} ,w_{i}^{{x_{{_{BL} }} }} ,w_{i}^{{z_{{_{BL} }} }} }} LF &= \sum\limits_{i = 1}^{m} {\frac{1}{{2(\sigma_{i}^{{hs_{TL} }} )^{2} }}} \left( {\mu_{i}^{{hs_{TL} }} (x_{i}^{{hs_{TL} }} - \varphi (y_{i} )w_{i}^{{x_{TL} }} - b_{i}^{{x_{TL} }} ) - (z_{i}^{{hs_{TL} }} - \varphi (y_{i} )w_{i}^{{z_{TL} }} - b_{i}^{{z_{TL} }} )} \right)^{2} \\ & \quad + \sum\limits_{i = 1}^{m} {\frac{1}{{2(\sigma_{i}^{{fs_{TL} }} )^{2} }}} \left( {\mu_{i}^{{fs_{TL} }} (z_{i}^{{fs_{TL} }} - \varphi (y_{i} )w_{i}^{{z_{TL} }} - b_{i}^{{z_{TL} }} ) - (x_{i}^{{fs_{TL} }} - \varphi (y_{i} )w_{i}^{{x_{TL} }} - b_{i}^{{x_{TL} }} )} \right)^{2} \\ & \quad + \sum\limits_{i = 1}^{m} {\frac{1}{{2(\sigma_{i}^{{hs_{BL} }} )^{2} }}} \left( {\mu_{i}^{{hs_{BL} }} (x_{i}^{{hs_{BL} }} - \varphi (y_{i} )w_{i}^{{x_{BL} }} - b_{i}^{{x_{BL} }} ) - (z_{i}^{{hs_{BL} }} - \varphi (y_{i} )w_{i}^{{z_{BL} }} - b_{i}^{{z_{BL} }} )} \right)^{2} \\ &\quad + \sum\limits_{i = 1}^{m} {\frac{1}{{2(\sigma_{i}^{{fs_{BL} }} )^{2} }}} \left( {\mu_{i}^{{fs_{BL} }} (z_{i}^{{fs_{BL} }} - \varphi (y_{i} )w_{i}^{{z_{BL} }} - b_{i}^{{z_{BL} }} ) - (x_{i}^{{fs_{BL} }} - \varphi (y_{i} )w_{i}^{{x_{BL} }} - b_{i}^{{x_{BL} }} )} \right)^{2}\end{aligned}}\\ &  s.t.\left\{ {\begin{array}{*{20}l} {\varphi (y_{i} )w_{i + 1}^{{z_{TL} }} + b_{i + 1}^{{z_{TL} }} \ge \varphi (y_{i} )w_{i}^{{z_{TL} }} + b_{i}^{{z_{TL} }} ,i = 1, \ldots ,{\text{k}} - 1} \hfill \\ {\varphi (y_{i} )w_{i + 1}^{{z_{BL} }} + b_{i + 1}^{{z_{BL} }} \ge \varphi (y_{i} )w_{i}^{{z_{BL} }} + b_{i}^{{z_{BL} }} ,i = 1, \ldots ,k - 1} \hfill \\ {\varphi (y_{i} )w_{i}^{{z_{TL} }} + b_{i}^{{z_{TL} }} \ge \varphi (y_{i} )w_{i}^{{z_{BL} }} + b_{i}^{{z_{BL} }} ,i = 1, \ldots ,{\text{k}}} \hfill \\ {\varphi (y_{1} )w_{1}^{{z_{BL} }} + b_{1}^{{z_{BL} }} \ge z_{\min }^{{fs_{j} }} } \hfill \\ {\varphi (y_{m} )w_{m}^{{z_{TL} }} + b_{m}^{{z_{TL} }} \le z_{\max }^{{fs_{j} }} } \hfill \\ \end{array} } \right., \\ \end{aligned} $$

where $(x_{i}^{{hs_{TL} }} ,y_{i}^{{}} ,z_{i}^{{hs_{TL} }} )$ and $(x_{i}^{{hs_{BL} }} ,y_{i}^{{}} ,z_{i}^{{hs_{BL} }} )$ represents the coordinate set of the end points of the middle level line of the i level plane in the upper and lower wall of the fault respectively. And $(x_{i}^{{fs_{TL} }} ,y_{i}^{{}} ,z_{i}^{{fs_{TL} }} )$ represents the set of coordinates corresponding to $(x_{i}^{{hs_{TL} }} ,y_{i}^{{}} ,z_{i}^{{hs_{TL} }} )$ in the fault line of the fault plane. In the same way, the $(x_{i}^{{fs_{BL} }} ,y_{i}^{{}} ,z_{i}^{{fs_{BL} }} )$ represents the set of coordinates corresponding to $(x_{i}^{{hs_{BL} }} ,y_{i}^{{}} ,z_{i}^{{hs_{BL} }} )$ in the fault line of the fault plane. $\mu_{i}^{{hs_{TL} }}$ and $\sigma_{i}^{{hs_{TL} }}$ represent the mean and standard deviation of the dip angle near the end point $(x_{i}^{{hs_{TL} }} ,y_{i}^{{}} ,z_{i}^{{hs_{TL} }} )$ of the hanging wall horizon line. $\mu_{i}^{{hs_{BL} }}$ and $\sigma_{i}^{{hs_{BL} }}$ represent the mean and standard deviation of the dip angle near the end point $(x_{i}^{{hs_{BL} }} ,y_{i}^{{}} ,z_{i}^{{hs_{BL} }} )$ of the hanging wall horizon line, $\mu_{i}^{{fs_{TL} }}$, $\sigma_{i}^{{fs_{TL} }}$ and $\mu_{i}^{{fs_{BL} }}$, $\sigma_{i}^{{fs_{BL} }}$ represent the mean and standard deviation of the reciprocal fault dip angle near fault points $(x_{i}^{{fs_{TL} }} ,y_{i}^{{}} ,z_{i}^{{fs_{TL} }} )$ and $(x_{i}^{{fs_{TL} }} ,y_{i}^{{}} ,z_{i}^{{fs_{TL} }} )$.

**Figure 12:**
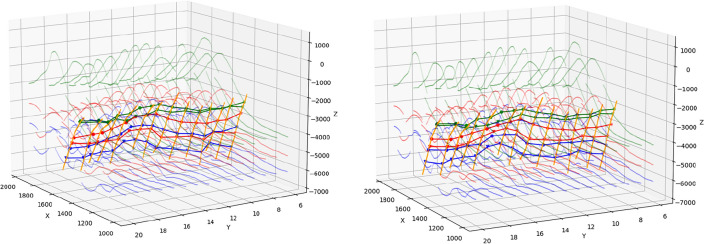
Comparison of boundary feature extraction methods. (**a**) Boundary characteristics guided by knowledge graph; (**b**) boundary characteristics of extrapolation interpolation.

By comparison in Fig. 12, it is found that in the traditional extrapolation interpolation method, the intersection lines of the second horizon and the first and third horizons are staggered. At the same time, considering that the fault is a reverse fault, the hanging-footwall intersection line is required to be located above the hanging-footwall intersection line. In the traditional extrapolation interpolation method, the intersecting lines of the upper and lower disks of the first horizon also appear interleaved. Under the guidance of the principle of knowledge graph, the method in this paper can expertly extract reliable boundary feature lines that are consistent with geological laws and expert cognition. Ensure that the intersection line of the deposited lower horizon always remains below the intersection line of the deposited upper horizon. Moreover, the hanging-wall (thrust fault) or hanging-wall (normal fault) intersection line always remains above the hanging-wall (thrust fault) or hanging-wall (normal fault) intersection line. This method effectively prevents the occurrence of intersecting lines of adjacent layers or intersecting lines of upper and lower layers, and eliminates any unreasonable phenomenon.

**Figure 13:**
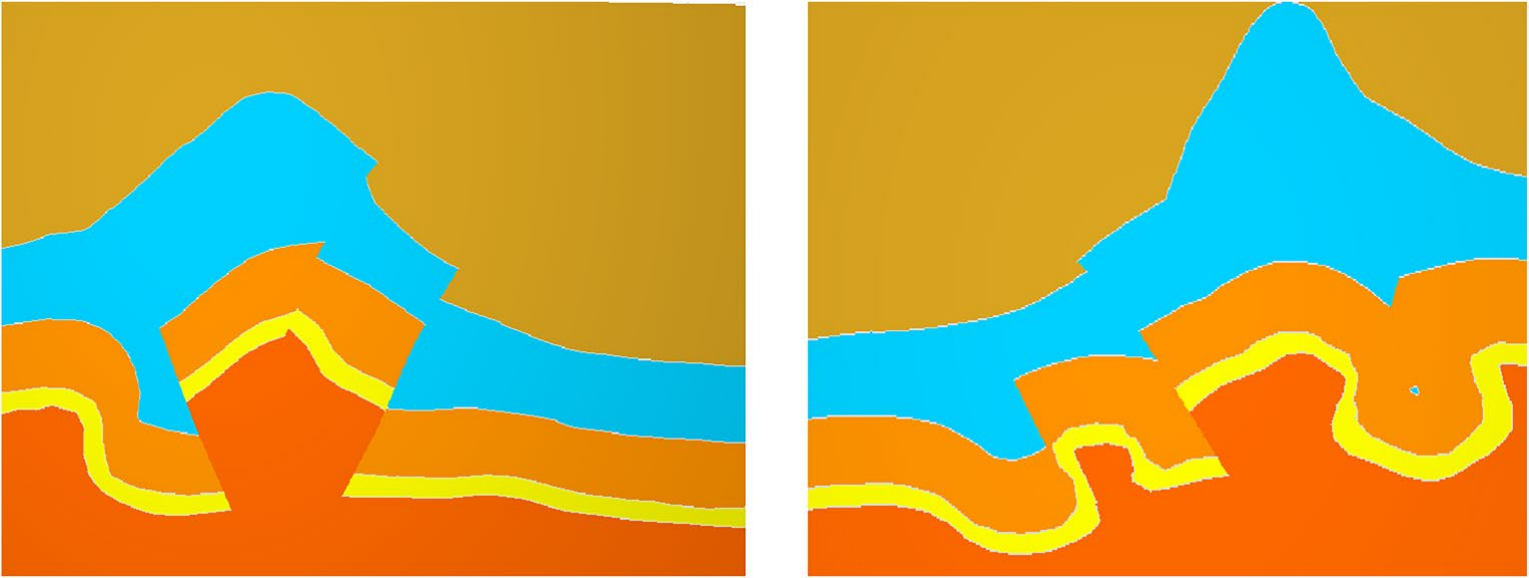
Three-dimensional geological structure (error).

**Figure 14:**
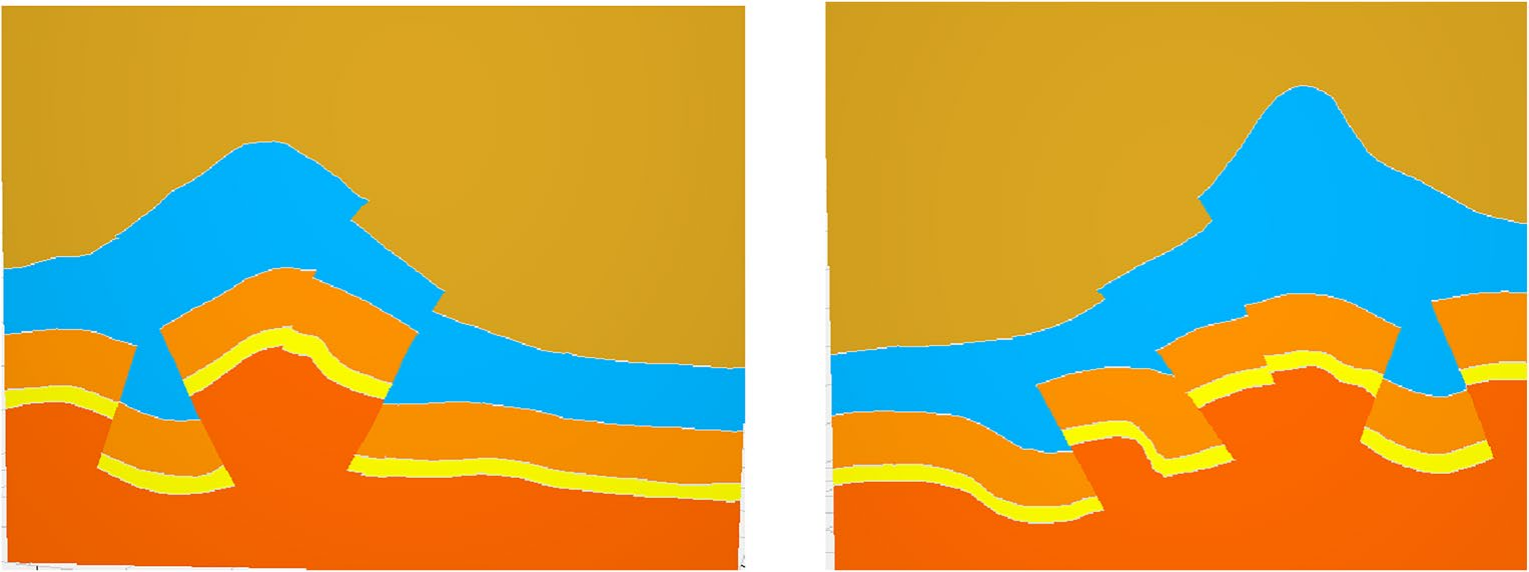
Three-dimensional geological structure.

To test the effectiveness of the complex geological knowledge graph based on knowledge reasoning, we constructed a three-dimensional geological model of the study area in Sichuan. When the traditional structural modeling method (Fig. 13) encounters an unreasonable fault or horizon, it obtains an accurate geological structural model by modifying the original data (Fig. 14). This method requires modification of a large amount of raw data, which is obviously not suitable for more complex work areas. But the method based on the knowledge reasoning (Fig. 10) can accurately constrain the three-dimensional geological modeling and reduce the uncertainty of structural modeling, what’s more, this method modifies the knowledge to model the constraints and avoids the uncertainty caused by modifying the original data.

### Other knowledge reasoning approaches

In addition, certain neural network-based methods, such as the graph neural network (GNN)^32,33^, can be used for subgraph isomorphism reasoning. These methods use the learning ability of neural networks to query whether a corresponding subgraph exists by learning the feature vectors of nodes and edges and describing the reasoning problem of the graph in detail.

Owing to the high requirements of domain knowledge graphs, ensuring the accuracy of results using a deep learning method is difficult if applied to practical engineering. The existing method constructs a model rule base by human definition to realize the construction of a knowledge graph; however, following the development of deep learning, the rules existing in the construction of knowledge graphs can be automatically learned, efficient knowledge reasoning can be realized, and the rule base can be avoided. For example, the hidden subface and closed block entities in a knowledge graph can be obtained by reasoning based on a random walk, and a topological semantic knowledge graph can be obtained. The AMIE algorithm proposed by Galárraga et al. is an association rule mining algorithm based on an incomplete knowledge base^34^. Each rule is predicted by learning: for each relationship, starting from the rule whose body is empty, the body part of the rule is expanded by three operations, and the candidate rules whose knowledge degree exceeds the threshold are retained to realize association rule mining research of knowledge graphs.

### Limitations

The method in this article aims to mine the topological structure of existing models from interpretation data. There is no better method for constructing topological semantic knowledge graphs for other data (such as seismic data); the limitation of this method is that it relies on Expert experience realizes the improvement of the knowledge reasoning rule base. For the limited work area data, our geological rule base can only build a knowledge graph and constrain the three-dimensional structural modeling for the existing work area. For other work areas, our method is theoretically applicable. However, for areas that are not accurate enough, we need to continuously add rules for reasoning to further improve our rule base. Based on a large number of experiments, our method is theoretically effective. Scalable. The disadvantage is that it relies on the participation of a large number of expert experience in the early stage, but in the actual work area, the accuracy of this method is relatively high and can meet the needs of actual production.

## Conclusions

This study introduces a technique for knowledge reasoning in the field of structural modeling, which can be applied to create modeling constraints for 3D geological structure modeling in the context of oil exploration. The knowledge graph we constructed enables experts and users to access the semantic information contained in the model at any time and maintain expert knowledge throughout the modeling process. Geological experts can visualize the knowledge graph more easily and find connections between knowledge items. The knowledge graph is used to constrain the boundary information of complex structural models, improving the efficiency of intelligent structural modeling.

## Data Availability

The datasets generated and/or analysed during the current study are not publicly available due [REASON WHY DATA ARE NOT PUBLIC] but are available from the corresponding author on reasonable request.
